# *Streptococcus thermophilus* DM287 and DM294 as candidate oral probiotic strains with anti-biofilm activity against cariogenic pathogens

**DOI:** 10.3389/fmicb.2026.1852167

**Published:** 2026-06-23

**Authors:** Jeong-Hoo Lee, Jia Yoo, Je-Hyun Eom, Young-Youn Kim, Hye-Sung Kim, Seung-Jo Yang

**Affiliations:** 1 R&D Center, DOCSMEDI Co., Ltd., Goyang, Republic of Korea; 2 Apple Tree Institute of Biomedical Science, Apple Tree Medical Foundation, Goyang, Republic of Korea; 3 Apple Tree Dental Hospital, Apple Tree Medical Foundation, Goyang, Republic of Korea

**Keywords:** biofilm inhibition, competitive colonization, dental caries, oral care ingredients, oral probiotics, *Streptococcus mutans*, *Streptococcus sobrinus*, *Streptococcus thermophilus*

## Abstract

*Streptococcus mutans* and *Streptococcus sobrinus* are key contributors to dental caries within a broader polymicrobial biofilm community, driving pathogenicity through biofilm and dental plaque formation rather than direct tissue invasion. Probiotic-based strategies that target colonization rather than viability represent a promising non-antibiotic approach to caries prevention. *Streptococcus thermophilus* is a food-grade organism with established GRAS status and a long history of safe human consumption, yet it remains largely unexplored as an oral care ingredient. In this study, two *S. thermophilus* strains – DM287, isolated from a commercially available yogurt product, and DM294, isolated from the tongue coat of healthy adults – were characterized for anti-cariogenic activity through mechanisms independent of major pathogen abundance reduction. Both strains reduced plaque mass by approximately 75%–80% in a wire model assay and suppressed *S. mutans* and *S. sobrinus* biofilm biomass to near-baseline levels, while planktonic growth suggested that the observed effects were not primarily driven by bactericidal activity. Adhesion inhibition assays showed 32%–64% reduction in pathogen adhesion under protection conditions and 72%–76% under displacement conditions. Competitive colonization assays demonstrated that both strains increased surface attachment in pathogen-conditioned environments (fold change > 1.0), consistent with competitive displacement behavior. RT-qPCR analysis of *S. mutans* biofilms co-cultured with either strain revealed significant downregulation of *gtfB* and *gtfC* – key glucosyltransferase genes encoding the EPS synthetic machinery central to biofilm structure – with reductions of approximately 34%–63% depending on the strain and time point, suggesting transcriptional suppression of biofilm-associated virulence as a contributing mechanism. EPS quantification by phenol–sulfuric acid assay further demonstrated that both strains reduced EPS production by 80%–90%, a magnitude comparable to chlorhexidine and substantially exceeding that of *Lacticaseibacillus rhamnosus* GG. Taken together, these findings suggest that *S. thermophilus* DM287 and DM294 are biocompatible candidate strains with promising anti-biofilm potential for oral care applications, pending further validation.

## 1 Introduction

Dental caries remains one of the most prevalent chronic infectious diseases globally, affecting an estimated 2.3 billion people with untreated carious lesions in permanent teeth (Qin et al., 2022). *Streptococcus mutans* and *Streptococcus sobrinus*, collectively referred to as mutans streptococci, are key contributors to dental caries within a broader polymicrobial biofilm community (Zheng et al., 2023). While dental caries is a polymicrobial, ecologically driven disease, these two species have been most extensively characterized as cariogenic pathogens and represent well-established targets for probiotic intervention research. Both species share key virulence traits that drive the cariogenic process: the production of glucosyltransferases (Gtfs) that synthesize extracellular polysaccharides (EPS) from dietary sucrose, enabling firm adhesion to the tooth surface and the subsequent formation of structured cariogenic biofilms (Rudin et al., 2023). The resulting dental plaque creates an acidogenic microenvironment that drives enamel demineralization, ultimately leading to cavitation. Critically, the pathogenicity of mutans streptococci depends less on invasive bactericidal interactions than on their capacity to colonize and form structured biofilms – making the inhibition of biofilm formation and surface attachment a strategically rational target for caries prevention (Zhu et al., 2023).

Conventional preventive strategies, including fluoride-containing dentifrices and chlorhexidine (CHX) mouthwash, exert their effects primarily through broad-spectrum antimicrobial activity (Nassar and Brizuela, 2023). While effective, these approaches carry well-documented limitations: fluoride toxicity concerns in young children, increasing evidence of CHX-associated disruption of the commensal oral microbiome, and the emergence of antimicrobial tolerance in oral streptococci with prolonged exposure (Iheozor-Ejiofor et al., 2024). These limitations have catalyzed interest in microbiome-modulating alternatives that selectively suppress cariogenic species without collateral disruption of the oral microbial ecosystem (Beattie, 2024).

Probiotic-based strategies offer a biologically coherent approach to this challenge. By introducing beneficial microorganisms capable of colonizing the oral cavity, probiotics can interfere with cariogenic pathogen adhesion and biofilm formation through competitive exclusion, co-aggregation-mediated displacement, and the remodeling of the local microenvironment through organic acid and bacteriocin production (Nizami et al., 2025). To date, research on oral probiotics has largely concentrated on *Lactobacillus* species – including *L. reuteri*, *L. rhamnosus*, and *L. paracasei* – and on *Streptococcus salivarius* strains K12 and M18, which have demonstrated clinical efficacy in reducing halitosis-associated volatile sulfur compounds and suppressing periodontal pathogens. By contrast, the functional role of food-derived lactic acid bacteria in the oral environment, particularly their potential to inhibit cariogenic biofilm formation, remains comparatively underexplored (Babina et al., 2023).

*Streptococcus thermophilus* is a representative food-grade lactic acid bacterium that has been widely used for decades in the manufacture of fermented dairy products including yogurt and certain cheeses, and its long history of safe consumption is reflected in its GRAS (Generally Recognized As Safe) status in the United States and Qualified Presumption of Safety (QPS) status in the European Union (Allouche et al., 2024). With an estimated annual human intake exceeding 10^21^ live cells, *S. thermophilus* is arguably among the most consumed microorganisms on the planet, and this exceptional safety record positions it as a compelling candidate for application beyond the food industry (Kapse et al., 2024). As a member of the salivarius phylogenetic subgroup – which includes the oral commensals *Streptococcus salivarius* and *Streptococcus vestibularis* – *S. thermophilus* shares evolutionary proximity with established oral probiotic species (Panel, 2022). Early *in vitro* evidence demonstrated that select *S. thermophilus* strains can adhere to saliva-coated hydroxyapatite beads and modulate the growth of cariogenic streptococci (Comelli et al., 2002), yet *S. thermophilus* remains only occasionally incorporated into oral care products and has rarely been characterized as a standalone oral care organism. A systematic, strain-level functional characterization of its cariostatic potential against the primary mutans streptococci–specifically *S. mutans* and *S. sobrinus*–has not been rigorously conducted within the context of dentifrice ingredient optimization (Banakar et al., 2024).

In the present study, we evaluated two *S. thermophilus* strains – DM287, isolated from a commercially available yogurt product, and DM294, isolated from the tongue coat of healthy adults – for their ability to suppress the biofilm formation and epithelial adhesion of cariogenic oral pathogens. Using a seven-assay *in vitro* panel encompassing macroscopic plaque quantification, biofilm biomass and planktonic growth assessment, epithelial adhesion inhibition, competitive colonization, cytotoxicity assessment in human oral epithelial cells, biofilm-associated virulence gene expression analysis, and comparative EPS quantification against reference controls, we provide a multidimensional functional characterization of these strains. Therefore, the present study aimed to determine whether two *S. thermophilus* strains of distinct ecological origin could suppress cariogenic biofilm formation and pathogen adhesion through non-bactericidal, EPS-targeting mechanisms relevant to oral probiotic applications.

## 2 Materials and methods

### 2.1 Bacterial strains and isolation

*Streptococcus thermophilus* DM287 was isolated from a commercially available yogurt product. Briefly, 1 g of yogurt was suspended in sterile phosphate-buffered saline (PBS), serially diluted, and plated onto M17 agar (KisanBio, Seoul, Korea) supplemented with 0.5% lactose. Plates were incubated at 37 °C for 24 h under anaerobic conditions, and morphologically distinct colonies were selected for purification. *Streptococcus thermophilus* DM294 was isolated from a healthy adult oral specimen distributed by the Appletree Oral Biobank (Apple Tree Dental Hospital, Goyang, Korea). Oral samples collected from 22 healthy adults were suspended in PBS, serially diluted, and plated onto M17 agar supplemented with 0.5% lactose. Plates were incubated at 37 °C for 24 h, and morphologically distinct colonies were selected for purification. Species identification was performed by 16S rRNA gene sequencing using universal primers 27F and 1492R. PCR amplicons were sequenced and queried against the NCBI BLAST database. Both strains exhibited ≥99% sequence identity to *Streptococcus thermophilus* reference sequences and were designated *S. thermophilus* DM287 and *S. thermophilus* DM294, respectively. Both strains were deposited at the Korean Collection for Type Cultures (KCTC) under accession numbers KCTC16622BP (DM287) and KCTC16550BP (DM294). Among the ten screening isolates (DM285–DM294) obtained from tongue coat specimens, one candidate (DM293) was excluded from further analysis after 16S rRNA sequencing identified it as *Lactiplantibacillus plantarum* (≈89%–90% identity to *S. thermophilus*). Pairwise sequence identity among all ten isolates is summarized in Supplementary Figure 1.

### 2.2 Bacterial culture conditions

*Streptococcus thermophilus* DM287 and DM294 were routinely cultured in M17 broth (KisanBio, Seoul, Korea) supplemented with 0.5% lactose at 37 °C for 18–24 h under anaerobic conditions. *Streptococcus mutans* KCOM1054 and *Streptococcus sobrinus* KCTC5273, used as cariogenic pathogen controls, were cultured in Brain Heart Infusion (BHI) broth (KisanBio, Seoul, Korea) at 37 °C under anaerobic conditions.

### 2.3 Wire model plaque assay

The inhibitory effect on plaque formation under simulated oral conditions was assessed using an orthodontic wire plaque model. *S. mutans* KCOM1054 or *S. sobrinus* KCTC5273 were inoculated into BHI broth supplemented with 0.5% lactose and 5% sucrose (pH 7.0, adjusted with 0.1 M MOPS) to a final concentration of approximately 1 × 10^8^ CFU/mL. *S. thermophilus* DM287 or DM294 were added simultaneously at an equivalent concentration. Probiotic and pathogen were co-inoculated simultaneously at matched cell densities (∼1 × 10^8^ CFU/mL each) to model a preventive co-colonization scenario, in which the probiotic and the cariogenic pathogen compete for the same surface from the outset, as would be relevant during daily dentifrice use. Sterile orthodontic stainless steel wires (0.016′′ diameter, 14′′ length; Ultimate Wireforms, USA) were submerged in the inoculated medium and incubated at 37 °C for 24 h. Following incubation, the resulting plaque was dried and weighed to quantify plaque mass.

### 2.4 Biofilm formation assay

Biofilm inhibition was evaluated by crystal violet staining. *S. mutans* KCOM1054 or *S. sobrinus* KCTC5273 were inoculated into BHI broth containing 0.5% lactose and 5% sucrose at approximately 1 × 10^6^–10^7^ CFU/mL in Corning Costar 96-well flat-bottom cell culture-treated microplates (Corning Inc., USA). *S. thermophilus* DM287 or DM294 were added simultaneously at approximately 5 × 10^7^ CFU/mL, and plates were incubated at 37 °C with 5% CO_2_ for 24 h. Supernatants were removed, wells were washed with PBS, and biofilms were stained with 0.1% crystal violet (DAEJUNG Chemicals & Metals Co., Ltd., Korea). After removal of excess stain and rinsing with distilled water, the stain was eluted with 95% ethanol and absorbance was measured at 590 nm using a microplate reader. Biofilm formation is expressed as relative biofilm formation (%) normalized to the untreated control.

### 2.5 Adhesion inhibition assay

The inhibitory effect of *S. thermophilus* DM287 and DM294 on cariogenic pathogen adhesion to oral epithelial cells was evaluated using human oral epithelial KB cells (Korean Cell Line Bank, No. 10017). KB cells were cultured in DMEM supplemented with 10% fetal bovine serum (FBS; Atlas Biologicals, USA) and 1% antibiotic–antimycotic solution (Corning, USA) at 37 °C with 5% CO_2_. Cells were seeded in 24-well plates at 2.5 × 105 cells/well and incubated for 24 h prior to bacterial exposure. Two experimental formats were employed. These two formats were designed to distinguish pre-emptive occupation of adhesion sites (protection) from active displacement of already-adhered pathogens (displacement). In the protection assay, KB cells were pre-treated with *S. thermophilus* DM287 or DM294 (1 × 10^8^ CFU/mL) for 1 h, washed twice with PBS, and then challenged with *S. mutans* KCOM1054 or *S. sobrinus* KCTC5273 (1 × 10^8^ CFU/mL) for 1 h. In the displacement assay, KB cells were first challenged with the pathogen (1 × 10^8^ CFU/mL) for 1 h, followed by addition of *S. thermophilus* DM287 or DM294 (1 × 10^8^ CFU/mL) for a further 1 h. Following incubation, both assays were processed under identical conditions: cells were washed twice with PBS to remove non-adherent bacteria, and the KB cell monolayers were lysed with TrypLE Express Enzyme (1×, no phenol red; Thermo Fisher Scientific, USA) to recover the adherent bacterial fraction. Genomic DNA was extracted from the recovered cells, and adherent pathogen abundance was quantified by species-specific quantitative real-time PCR (qPCR) using SYBR Green chemistry, with relative pathogen adhesion calculated from cycle threshold (Ct) values normalized to the pathogen-only control.

### 2.6 Competitive colonization assay

The capacity of *S. thermophilus* strains to competitively displace pre-adhered cariogenic pathogens was assessed. *S. mutans* KCOM1054 or *S. sobrinus* KCTC5273 were first adhered to KB cells as described above. *S. thermophilus* DM287 or DM294 were subsequently added and co-incubated under the same conditions. Cells were washed and adherent bacteria were recovered. Genomic DNA was extracted and pathogen abundance was quantified by qPCR. Results are expressed as fold change relative to the pathogen-only control.

### 2.7 Cytotoxicity assays

The cytotoxicity of *S. thermophilus* DM287 and DM294 toward KB human oral epithelial cells was evaluated using two complementary assays – lactate dehydrogenase (LDH) release for membrane integrity and 3-(4,5-dimethylthiazol-2-yl)-2,5-diphenyltetrazolium bromide (MTT) reduction for metabolic viability – under both heat-killed and live bacterial treatment conditions. For the LDH release assay (Quanti-LDH PLUS Cytotoxicity Assay Kit, Colorimetric; ScienCell, USA), KB cells were seeded in 96-well plates at 1 × 10^4^ cells/well in RPMI-1640 medium supplemented with 10% FBS at 37 °C with 5% CO_2_ for 24 h. The medium was then replaced with antibiotic-free RPMI-1640 supplemented with 10% FBS, and cells were treated for 24 h with either heat-killed *S. thermophilus* DM287 or DM294 (1 × 10^8^ CFU/mL equivalent) or live *S. thermophilus* DM287 or DM294 (MOI 200). Following incubation, 50 μL of supernatant from each well was transferred to a new plate, mixed with 50 μL of LDH reaction solution, and incubated in the dark for 30 min. Absorbance was measured at 490 nm, and cytotoxicity (%) was calculated relative to a maximum LDH release control (cell lysate; high control) and a spontaneous LDH release control (untreated cells; low control). For the MTT assay, KB cells were seeded in 96-well plates at 5 × 10^4^ cells/well in RPMI-1640 medium supplemented with 10% FBS and incubated for 24 h, after which the medium was replaced with antibiotic-free RPMI-1640 supplemented with 10% FBS. Cells were then treated for 24 h with either heat-killed or live *S. thermophilus* DM287 or DM294 under the same density conditions described above, washed with PBS, and incubated with MTT solution (final concentration 0.5 mg/mL) at 37°C for 4 h. Supernatants were removed, formazan crystals were dissolved in 100 μL DMSO, and absorbance was measured at 570 nm after 15 min at room temperature. Cell viability (%) was expressed relative to the untreated low control.

### 2.8 Biofilm-related gene expression analysis

The effect of *S. thermophilus* DM287 and DM294 on the expression of biofilm-associated virulence genes in *S. mutans* was evaluated by quantitative real-time PCR (qPCR). *S. mutans* KCOM1054 was inoculated into BHI broth supplemented with 5% sucrose at approximately 1 × 10^6^ CFU/mL. *S. thermophilus* DM287 or DM294 was added simultaneously at approximately 1 × 10^6^–10^7^ CFU/mL, and cultures were incubated under static conditions at 37 °C. Biofilm samples were collected at 8 h (early biofilm stage) and 24 h (mature biofilm stage). Following removal of the supernatant and two washes with PBS to eliminate planktonic cells, total RNA was extracted from the collected biofilm using the illustra™ RNAspin Mini RNA Isolation Kit (Cytiva, USA). Complementary DNA was synthesized using the PrimeScript™ RT Reagent Kit (Takara Bio, Japan). Real-time PCR was performed on a QuantStudio™ 3 Real-Time PCR System (Applied Biosystems, USA) using TOPreal™ qPCR 2 × PreMIX (Enzynomics, Korea) for SYBR Green-based detection. Thermal cycling conditions were as follows: 95 °C for 10 min, followed by 40 cycles of 95 °C for 10 s, 60 °C for 15 s, and 72 °C for 20 s. Fluorescence data were collected at 72 °C, and melt curve analysis was conducted to verify amplification specificity. The expression of glucosyltransferase B (*gtfB*) and glucosyltransferase C (*gtfC*), key virulence genes involved in sucrose-dependent biofilm formation, was quantified. Primer sequences were as follows: *gtfB* (forward: 5′-AGCAATGCAGCCATCTACAAAT-3′, reverse: 5′-ACGAACTTTGCCGTTATTGTCA-3′) and *gtfC* (forward: 5′-CTCAACCAACCGCCACTGTT-3′, reverse: 5′-GGTTTAACGTCAAAATTAGCTGTATTAGC-3′) (Ahn et al., 2005). *gyrA* was used as the housekeeping reference gene (forward: 5′-CCAAGAATCTGCTGTCCG-3′, reverse: 5′-TTGCGACTATCTGCTATGTG-3′) (He et al., 2017). Relative gene expression was calculated using the 2^∧^(−ΔΔCt) method, normalized to the *S. mutans*-only control.

To further assess whether biofilm suppression was associated with reduced pathogen abundance, species-specific qPCR was additionally performed using genomic DNA extracted from co-culture samples. Total bacterial cells were collected from the entire culture, and genomic DNA (gDNA) was extracted using the Bacteria Genomic DNA Isolation Kit (LaboPass, COSMO Genetech, Seoul, Korea) according to the manufacturer’s instructions. For *S. mutans* abundance analysis, the same *gtfB* primer set used for RT-qPCR analysis was applied to genomic DNA samples. *S. sobrinus* abundance was quantified using *S. sobrinus*-specific primers. Relative abundance values were calculated using the 2^∧^(−ΔΔCt) method and normalized to the corresponding pathogen-only control group.

Primer sequences were as follows: *S. mutans* gtfB (forward: 5′-AGCAATGCAGCCATCTACAAAT-3′, reverse: 5′-ACGAACTTTGCCGTTATTGTCA-3′) and *S. sobrinus* gtfI (forward: 5′-GCCAGCGATACACTCTTCTTA-3′, reverse: 5′-TGACTTGGTCACCAGTTGCA-3′).

### 2.9 Comparative biofilm inhibition and EPS quantification

To compare the anti-biofilm efficacy of DM287 and DM294 with established reference controls and to determine whether suppression of gtfB and gtfC expression was associated with reduced extracellular polysaccharide (EPS) production, additional experiments were performed using *S. mutans* KCOM1054, as sucrose-dependent EPS synthesis mediated by glucosyltransferases is most extensively characterized in this species.

*Lacticaseibacillus rhamnosus* GG (LGG; ATCC 53103), a commercially utilized probiotic reference strain, was routinely cultured in MRS broth (KisanBio, Seoul, Korea) at 37 °C for 18–24 h under anaerobic conditions prior to use.

*Streptococcus mutans* was inoculated into BHI broth containing 0.5% lactose and 5% sucrose at approximately 1 × 10^6^–10^7^ CFU/mL in SPL 24-well flat-bottom plates (SPL Life Sciences, Pocheon, Republic of Korea). *S. thermophilus* DM287 or DM294, or LGG, were added simultaneously at approximately 5 × 10^7^ CFU/mL. Chlorhexidine digluconate (Hexamedine solution 0.12%; Bukwang Pharm., Seoul, Republic of Korea) was added simultaneously at a final concentration of 0.005% (v/v) as a positive control. All conditions were incubated at 37 °C with 5% CO_2_ for 24 h. Biofilm biomass was quantified by crystal violet staining as described in Section “2.4 Biofilm formation assay,” with absorbance measured at OD_590_.

For EPS quantification, following removal of culture supernatants and PBS washing, biofilm-associated EPS was extracted using 0.5 M NaOH and quantified using the phenol–sulfuric acid method. Absorbance was measured at 490 nm using a microplate reader, and relative EPS production (%) was expressed relative to the *S. mutans*-only control group.

### 2.10 Ethics statement

The use of human oral specimens for the isolation of *S. thermophilus* DM294 was conducted in accordance with the ethical standards of the institutional review board. This study received IRB exemption confirmation from Apple Tree Dental Hospital (No. P01-202508-02-012). Bacterial strains were obtained through the Appletree Oral Biobank under an approved distribution agreement (No. 25082601-48-01). As specimens were distributed through an institutional biobank under established oversight protocols, no additional informed consent was required.

### 2.11 Statistical analysis

Statistical analyses were performed using GraphPad Prism (version 9; GraphPad Software, USA), with confirmatory analyses conducted in Python (SciPy v1.10.0). Data are presented as mean ± standard deviation (SD) with individual data points overlaid. Sample sizes differed by assay: the biofilm formation assay (Figure 2) was performed as two independent experiments each in triplicate (*n* = 6 per group), whereas all other assays including the comparative biofilm inhibition and EPS quantification assays (Figure 6) were performed in three independent replicates (*n* = 3 per group). A *p*-value < 0.05 was considered statistically significant throughout.

**FIGURE 1:**
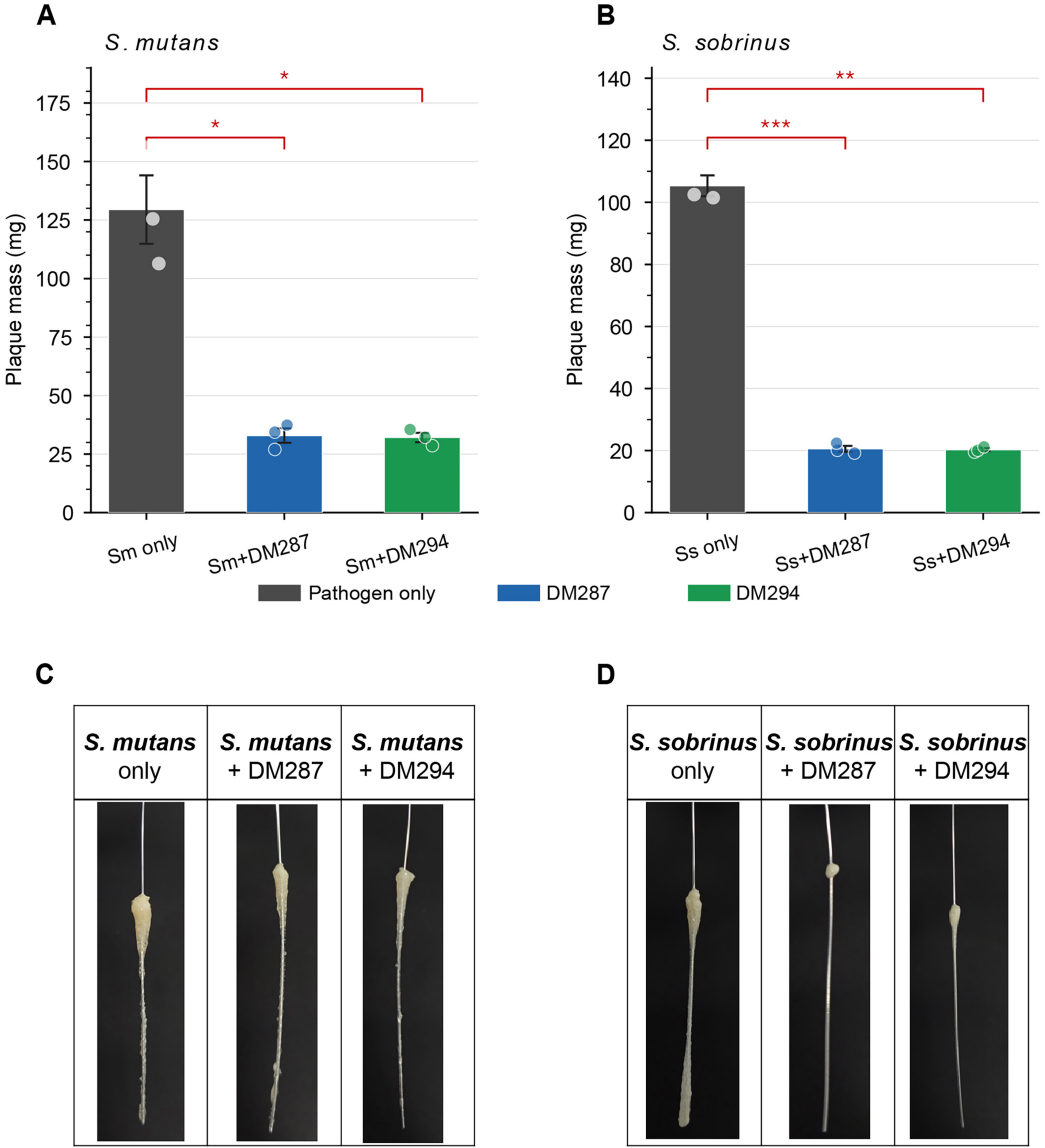
Inhibition of cariogenic plaque formation by *S. thermophilus* DM287 and DM294 in a wire model plaque assay. **(A)** Plaque mass (mg) formed on orthodontic stainless steel wires after 24 h co-culture of *S. mutans* with *S. thermophilus* DM287 or DM294 at matched cell densities (∼1 × 10^8^ CFU/mL each) in BHI broth supplemented with 0.5% lactose and 5% sucrose (pH 7.0). **(B)** Plaque mass (mg) formed under the same conditions using *S. sobrinus* as the cariogenic pathogen. **(C)** Representative photographs of orthodontic wire specimens following *S. mutans* plaque formation under pathogen-only, DM287 co-culture, and DM294 co-culture conditions. **(D)** Representative photographs of orthodontic wire specimens following *S. sobrinus* plaque formation under the same conditions. Bars represent mean ± standard deviation (SD) of triplicate measurements; individual data points are overlaid. Statistical significance was assessed by one-way ANOVA followed by Dunnett’s multiple comparison test against the respective pathogen-only control [*S. mutans* for panels **(A,C)**; *S. sobrinus* for panels **(B,D)**]. *p < 0.05; **p < 0.01; ***p < 0.001.

**FIGURE 2:**
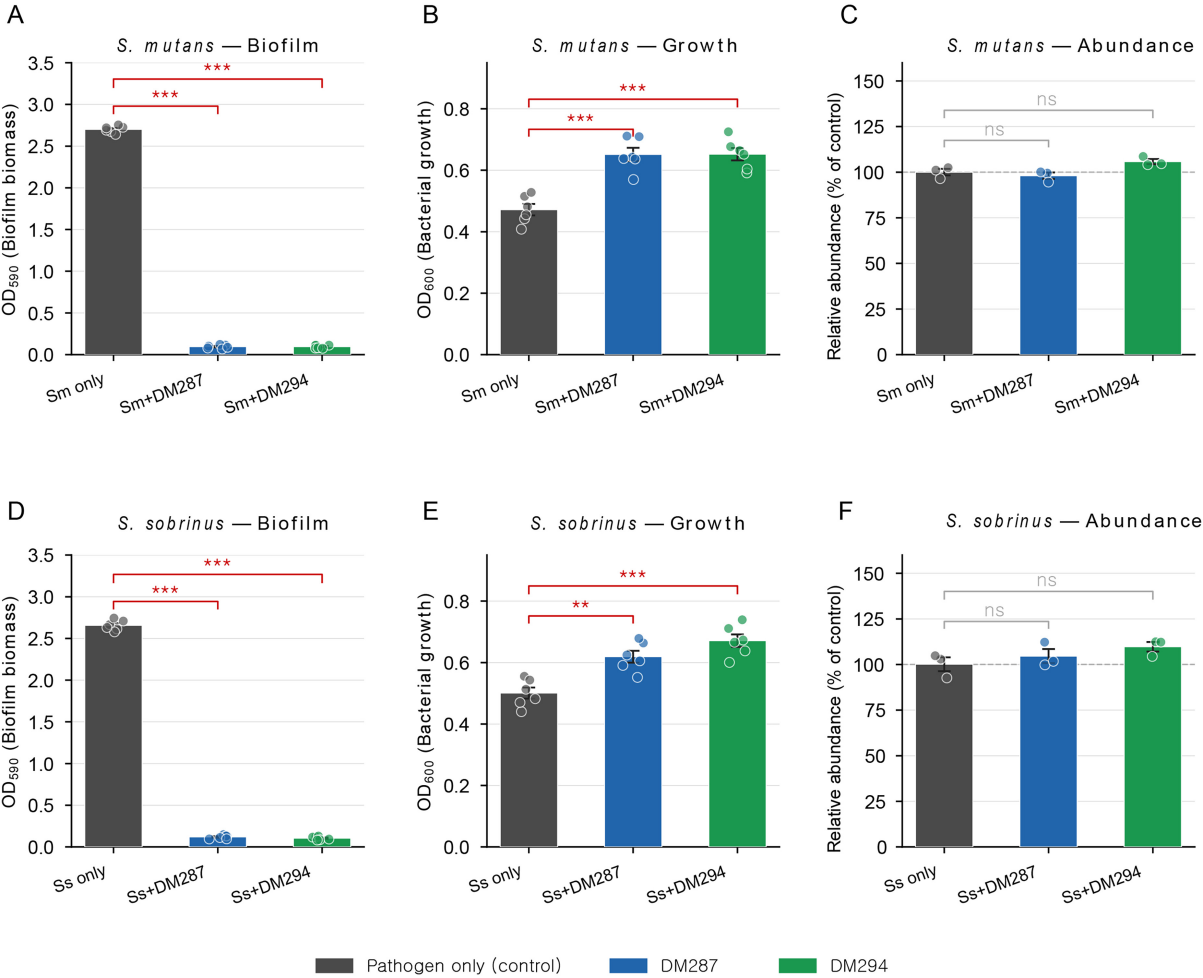
Suppression of cariogenic biofilm biomass without substantial reduction in pathogen abundance by *S. thermophilus* DM287 and DM294. **(A)** Biofilm biomass (OD_590_) of *S. mutans* quantified by crystal violet staining after 24 h co-culture with *S. thermophilus* DM287 or DM294 (∼5 × 10^7^ CFU/mL each) in BHI broth supplemented with 5% sucrose and 0.5% lactose. **(B)** Planktonic bacterial growth (OD600) measured in co-culture supernatants for *S. mutans*. **(C)** Relative abundance of *S. mutans* under co-culture conditions quantified by species-specific qPCR and normalized to the *S. mutans*-only control group. **(D)** Biofilm biomass (OD_590_) of *S. sobrinus* quantified under the same conditions. **(E)** Planktonic bacterial growth (OD600) measured in co-culture supernatants for *S. sobrinus*. **(F)** Relative abundance of *S. sobrinus* under co-culture conditions quantified by species-specific qPCR and normalized to the *S. sobrinus*-only control group. Bars represent mean ± SD; *n* = 6 per group (two independent experiments, each performed in triplicate). Individual data points are overlaid. Statistical significance was assessed by one-way ANOVA followed by Dunnett’s multiple comparison test against the respective pathogen-only control group. ns, not significant. **p < 0.01; ***p < 0.001.

**FIGURE 3:**
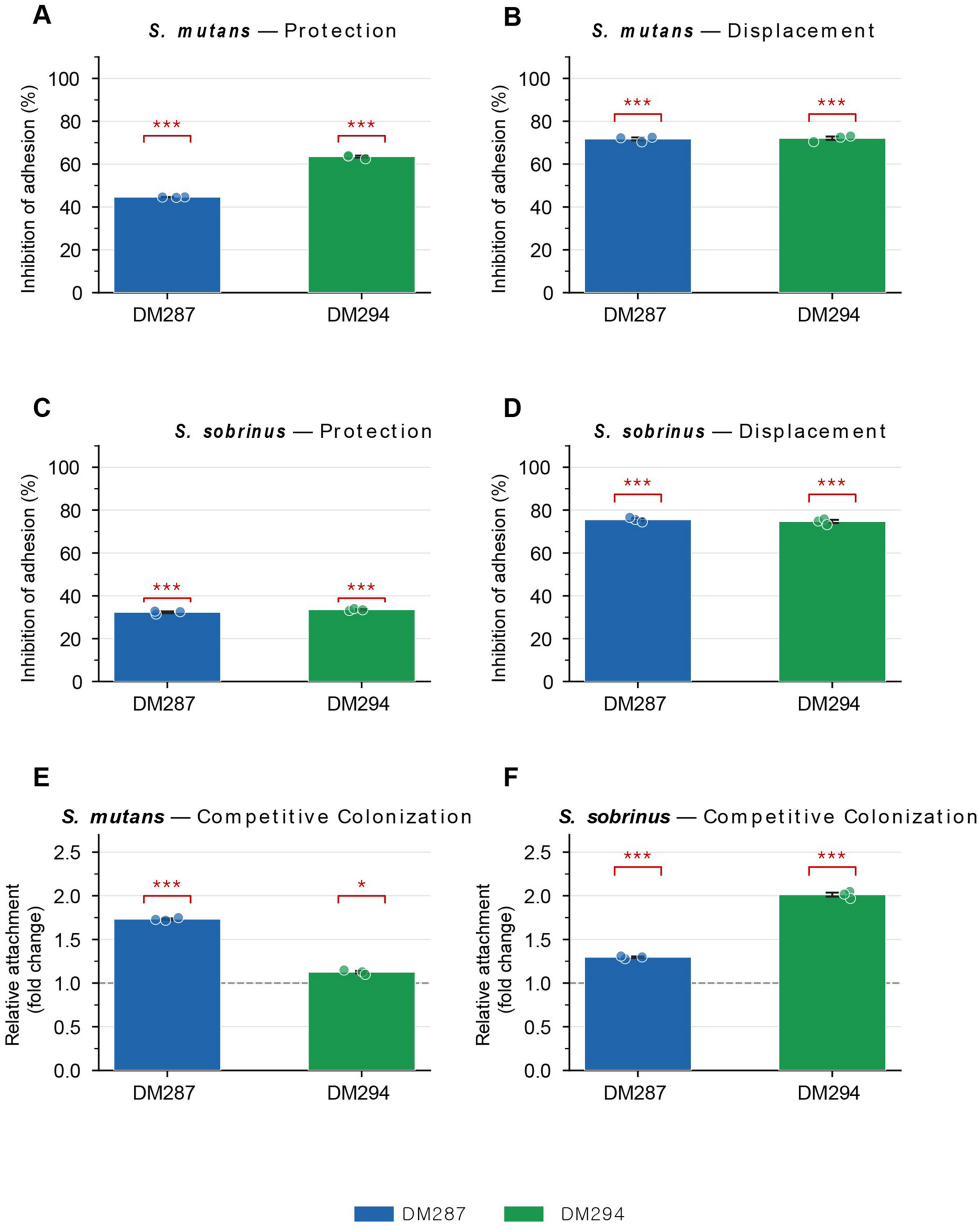
Inhibition of pathogen adhesion **(A–D)** and competitive colonization in pathogen-conditioned environments **(E,F)** on KB oral epithelial cells by *S. thermophilus* DM287 and DM294. **(A)** Protection assay against *S. mutans*: KB cell monolayers were pre-treated with *S. thermophilus* DM287 or DM294 (1 × 10^8^ CFU/mL) for 1 h prior to pathogen challenge for a further 1 h. **(B)** Displacement assay against *S. mutans*: the pathogen was first allowed to adhere for 1 h, followed by introduction of *S. thermophilus* DM287 or DM294 for 1 h. **(C)** Protection assay against *S. sobrinus*, conducted under the same conditions as described for panel **(A)**. **(D)** Displacement assay against *S. sobrinus*, conducted under the same conditions as described for panel **(B)**. For panels **(A–D)**, non-adherent bacteria were removed by PBS washing, adherent pathogen load was quantified by species-specific qPCR (SYBR Green), and adhesion inhibition (%) was calculated relative to the pathogen-only control. **(E)** Competitive colonization against *S. mutans*: pre-attachment of *S. mutans* (1 × 10^8^ CFU/mL) for 1 h was followed by *S. thermophilus* DM287 or DM294 (1 × 10^8^ CFU/mL) for a further 1 h. **(F)** Competitive colonization against *S. sobrinus*, conducted under the same conditions as described for panel **(E)**. For panels **(E,F)**, adherent *S. thermophilus* was recovered by TrypLE-mediated cell lysis and quantified by species-specific qPCR; fold change was normalized to the respective *S. thermophilus*-alone condition (dashed line = 1.0). Bars represent mean ± standard deviation (SD); *n* = 3 per group. Individual data points are overlaid. Statistical significance was assessed by one-sample *t*-test against a null value of 0% for panels **(A–D)** and against a null value of 1.0 for panels **(E,F)**. *p < 0.05; ***p < 0.001.

**FIGURE 4:**
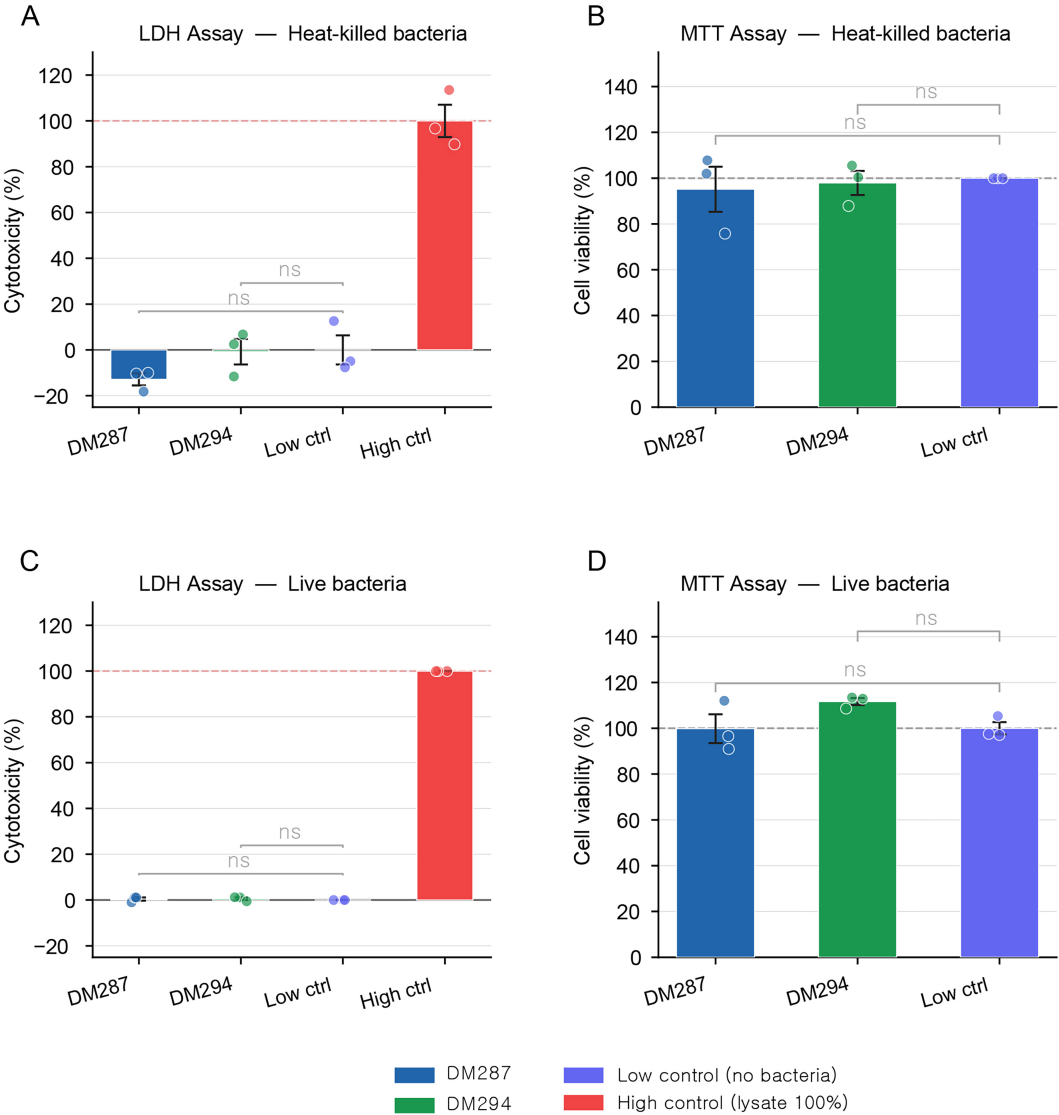
Cytotoxicity of *S. thermophilus* DM287 and DM294 toward KB human oral epithelial cells. **(A)** LDH release assay measuring cell membrane integrity. Cytotoxicity (%) was calculated relative to a maximum LDH release control (high control; 100% cell lysate) and a spontaneous LDH release control (low control; untreated cells). The dashed red line indicates the 100% cytotoxicity reference. **(B)** MTT assay measuring metabolic activity. **(C)** LDH assay and **(D)** MTT assay following treatment with live *S. thermophilus* DM287 and DM294 (MOI 200) under identical experimental conditions. Cell viability (%) was normalized to untreated cells (low control = 100%). The dashed gray line indicates the 100% viability reference. KB cells were seeded at 1 × 10^4^ cells/well (LDH assay) or 5 × 10^4^ cells/well (MTT assay) and treated with heat-killed or live *S. thermophilus* DM287 or DM294 (1 × 10^8^ CFU/mL equivalent) for 24 h at 37 °C with 5% CO_2_. Bars represent mean ± standard deviation (SD); *n* = 3 per group. Individual data points are overlaid. Statistical significance was assessed by one-sided Dunnett’s multiple comparison tests against the untreated low control, with the alternative hypothesis directionally specified to match the biological readout (LDH release: alternative = “greater”; MTT viability: alternative = “less”). ns, not significant (*p* ≥ 0.05).

**FIGURE 5:**
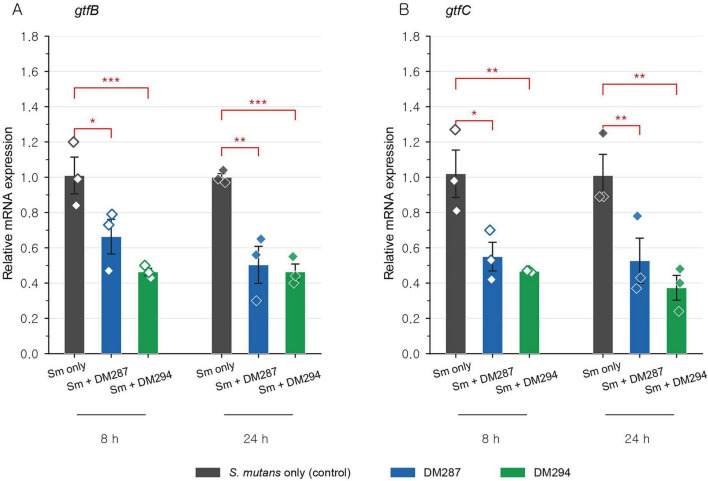
Downregulation of *gtfB* and *gtfC* expression in *S. mutans* biofilms by *S. thermophilus* DM287 and DM294. **(A)** Relative mRNA expression of *gtfB* at 8 h and 24 h in *S. mutans* biofilms co-cultured with *S. thermophilus* DM287 or DM294. **(B)** Relative mRNA expression of *gtfC* at 8 h and 24 h under the same conditions. *S. mutans* KCOM1054 (∼1 × 10^6^ CFU/mL) was co-cultured with *S. thermophilus* DM287 or DM294 (∼1 × 10^6^–10^7^ CFU/mL) in BHI broth supplemented with 0.5% lactose and 5% sucrose under static conditions at 37 °C. Biofilm samples were collected at 8 h and 24 h. Total RNA was extracted from washed biofilms, and gene expression was quantified by RT-qPCR using *gyrA* as the housekeeping reference gene. Relative expression levels were calculated using the 2^∧^(–ΔΔCt) method and normalized to the *S. mutans*-only control. Bars represent mean ± standard deviation (SD) with individual data points overlaid (*n* = 3 per group). Statistical significance was assessed by two-way ANOVA with treatment and time as factors, followed by Dunnett’s multiple comparison test against the *S. mutans*-only control within each time point. *p < 0.05; **p < 0.01; ***p < 0.001.

**FIGURE 6:**
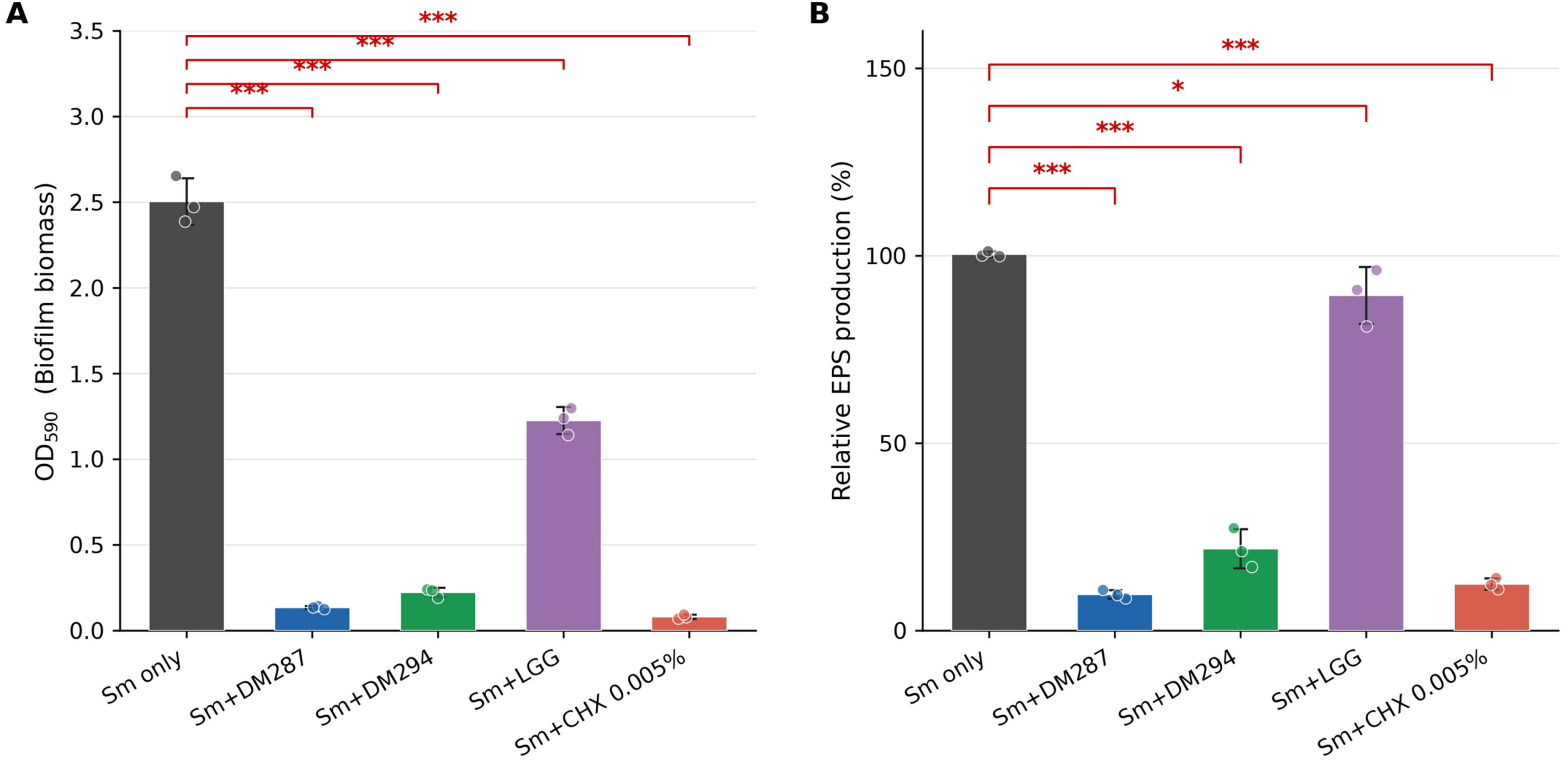
Comparative inhibition of *S. mutans* biofilm formation and extracellular polysaccharide (EPS) production by *S. thermophilus* DM287 and DM294. **(A)** Biofilm biomass of *S. mutans* following 24 h co-culture with *S. thermophilus* DM287, DM294, *Lacticaseibacillus rhamnosus* GG (LGG), or chlorhexidine (CHX; 0.005%) in BHI broth supplemented with 0.5% lactose and 5% sucrose, quantified by crystal violet staining (OD_590_). **(B)** Relative EPS production in *S. mutans* biofilms under the same co-culture conditions. Biofilm-associated EPS was extracted using 0.5 M NaOH and quantified by the phenol–sulfuric acid colorimetric method (OD_490_). All values were normalized to the *S. mutans*-only control group. Bars represent mean ± SD; *n* = 3 per group. Statistical significance was assessed by one-way ANOVA followed by Dunnett’s multiple comparison test against the *S. mutans*-only control. *p < 0.05; ***p < 0.001.

For the plaque mass and biofilm formation assays (Figures 1, 2), differences between groups were assessed by one-way ANOVA followed by Dunnett’s multiple comparison test against the respective pathogen-only control. For the adhesion inhibition assay (Figures 3A–D), inhibition rates were compared against a null value of 0% by one-sample *t*-test. For the competitive colonization assay (Figures 3E, F), fold-change values were compared against a null value of 1.0 by one-sample *t*-test. For the cytotoxicity assays (Figure 4), one-sided Dunnett’s multiple comparison tests were applied against the untreated low control, with the alternative hypothesis directionally specified to match the biological readout (LDH release: alternative = “greater”; MTT viability: alternative = “less”); this directional framing reflects that elevated LDH release and reduced MTT signal are the only biologically interpretable indicators of cytotoxicity. For the biofilm-related gene expression analysis (Figure 5), data were analyzed by two-way ANOVA with treatment and biofilm maturation time as factors, including the treatment × time interaction term, followed by Dunnett’s multiple comparison test (using the pooled mean square error from the two-way ANOVA) comparing each treatment with the *S. mutans*-only control within each time point. For the comparative biofilm inhibition and EPS quantification assays (Figure 6), differences between groups were assessed by one-way ANOVA followed by Dunnett’s multiple comparison test against the *S. mutans*-only control.

## 3 Results

### 3.1 Reduction of cariogenic plaque formation on orthodontic wire surfaces

A wire model plaque assay was performed to evaluate the ability of *S. thermophilus* DM287 and DM294 to suppress dental plaque formation under simulated oral conditions. In the absence of probiotic strains, *S. mutans* and *S. sobrinus* formed substantial plaque deposits on orthodontic wire with mean plaque masses of 129.5 ± 14.6 mg and 105.3 ± 3.3 mg, respectively (Figures 1A,B). Co-culture with either *S. thermophilus* strain markedly reduced plaque accumulation by both pathogens. Against *S. mutans*, DM287 and DM294 reduced mean plaque mass to 32.9 ± 3.1 mg (74.6% reduction; *p* < 0.05) and 32.1 ± 2.0 mg (75.2% reduction; *p* < 0.05), respectively (Figure 1A). Against *S. sobrinus*, reductions were even more pronounced: 20.6 ± 1.0 mg with DM287 (80.5%; *p* < 0.001) and 20.3 ± 0.5 mg with DM294 (80.7%; *p* < 0.01) (Figure 1B). The two strains performed comparably against each pathogen, with no significant difference in plaque reduction between them. Visual inspection of the wire surfaces was consistent with the gravimetric findings, showing dense, cohesive deposits in pathogen-only conditions and markedly sparse plaque in co-cultured specimens (Figures 1C,D).

### 3.2 Non-bactericidal interference with cariogenic biofilm development

To clarify the mechanism behind plaque inhibition, biofilm biomass and planktonic growth were measured simultaneously in 96-well co-culture assays using crystal violet staining (OD_590_) and optical density (OD600), respectively. Both strains produced profound inhibition of biofilm biomass for both pathogens. *S. mutans* biofilm OD_590_ decreased from 2.70 ± 0.02 in the pathogen-only condition to 0.096 ± 0.009 with DM287 and 0.097 ± 0.007 with DM294 (both 96.4% reduction; *p* < 0.001) (Figure 2A). *S. sobrinus* biofilm OD_590_ similarly fell from 2.66 ± 0.03 to 0.120 ± 0.009 with DM287 (95.5%; *p* < 0.001) and 0.10^6^ ± 0.007 with DM294 (96.0%; *p* < 0.001) (Figure 2D). Despite this striking reduction in biofilm, planktonic bacterial density in co-culture supernatants was not reduced relative to pathogen-only controls – in fact, OD600 values were significantly higher in all co-culture conditions, likely reflecting, at least in part, the additive contribution of *S. thermophilus* cells to total planktonic density. *S. mutans* OD600 increased from 0.472 ± 0.019 to 0.651 ± 0.021 with DM287 and 0.652 ± 0.019 with DM294 (both *p* < 0.001) (Figure 2B), while *S. sobrinus* OD600 rose from 0.501 ± 0.018 to 0.619 ± 0.018 with DM287 (*p* < 0.01) and 0.672 ± 0.018 with DM294 (*p* < 0.001) (Figure 2E). These findings indicate that neither DM287 nor DM294 markedly reduces total planktonic bacterial density under the tested conditions.

Although OD600 measurements reflect total bacterial density and cannot distinguish between individual species in co-culture systems, the lack of reduction in overall OD600 suggested that the observed anti-biofilm effect was unlikely to be primarily driven by bactericidal activity. Species-specific qPCR further demonstrated that the relative abundance of both *S. mutans* and *S. sobrinus* was not significantly altered under co-culture conditions with DM287 or DM294 (Figures 2C,F), despite marked suppression of biofilm biomass. These findings support the interpretation that the observed anti-biofilm activity was not primarily attributable to reduced pathogen abundance.

### 3.3 Inhibition of pathogen adhesion via preventive and competitive modes

The ability of *S. thermophilus* DM287 and DM294 to interfere with pathogen adhesion to oral epithelial cells was assessed using KB cells under two conditions: a protection assay, in which the probiotic was applied before pathogen exposure, and a displacement assay, in which it was applied after pathogen pre-attachment. In the protection assay, pre-treatment of KB cells with either strain significantly reduced subsequent *S. mutans* adhesion: DM287 achieved 44.6% ± 0.1% inhibition and DM294 achieved 63.5% ± 0.51% inhibition (both *p* < 0.001), with DM294 showing notably greater efficacy (Figure 3A). Against *S. sobrinus*, protective inhibition was more modest at 32.3% ± 0.42% for DM287 and 33.6% ± 0.26% for DM294 (both *p* < 0.001) (Figure 3C). In the displacement assay, inhibition was substantially higher across all conditions. *S. mutans* adhesion was reduced by 71.8% ± 0.68% with DM287 and 72.1% ± 0.80% with DM294 (both *p* < 0.001) (Figure 3B), and *S. sobrinus* adhesion by 75.6% ± 0.61% and 74.7% ± 0.78%, respectively (both *p* < 0.001) (Figure 3D). The consistently higher inhibition in the displacement condition – most evident against *S. sobrinus* (∼75% vs. ∼33%) – suggests that both strains are capable of actively dislodging already-adhered pathogens from the epithelial surface, not merely occupying adhesion sites in advance.

### 3.4 Enhanced surface affinity and competitive displacement capacity

Competitive colonization assays were performed to determine whether *S. thermophilus* DM287 and DM294 can colonize epithelial surfaces already occupied by cariogenic pathogens. Probiotic attachment was expressed as fold change relative to the probiotic-alone condition (baseline = 1.0), where values significantly above 1.0 indicate active competitive colonization. In all four strain–pathogen combinations, probiotic attachment was significantly elevated in pathogen-conditioned environments. Against *S. mutans*, DM287 attachment increased to 1.73 ± 0.009-fold (*p* < 0.001) and DM294 to 1.13 ± 0.015-fold (*p* < 0.05) (Figure 3E). Against *S. sobrinus*, DM287 reached 1.30 ± 0.009-fold (*p* < 0.001) and DM294 showed the strongest competitive response at 2.01 ± 0.023-fold (*p* < 0.001) (Figure 3F). The two strains showed opposite patterns of competitive affinity: DM287 competed more effectively against *S. mutans* (1.73-fold) than *S. sobrinus* (1.30-fold), while DM294 showed considerably stronger competition against *S. sobrinus* (2.01-fold) than *S. mutans* (1.13-fold), indicating strain-specific differences in competitive colonization behavior across the two pathogens.

### 3.5 Cytotoxicity in human oral epithelial cells

To evaluate the safety of *S. thermophilus* DM287 and DM294 for topical oral care applications, cytotoxicity was first assessed using heat-killed bacterial preparations in LDH release and MTT assays on KB human oral epithelial cells (Figures 4A,B). Neither strain induced measurable LDH release; cytotoxicity values were −12.9% ± 4.6% for DM287 and −0.8% ± 9.6% for DM294, both statistically indistinguishable from the untreated low control (*p* = ns) (Figure 4A). The slightly negative value for DM287 reflects background absorbance variation inherent to the LDH assay rather than any biologically meaningful effect, and the high control (∼100% LDH release) confirmed that the assay performed as expected. Cell viability in the MTT assay was similarly unaffected, with mean values of 95.2% ± 17.0% for DM287 and 97.9% ± 9.1% for DM294, neither of which differed significantly from untreated cells (*p* = ns) (Figure 4B). The wider spread in the DM287 group is attributable to one outlier replicate (75.8%) and was not indicative of a cytotoxic effect. These findings indicate that heat-killed *S. thermophilus* DM287 and DM294 are well tolerated by oral epithelial cells at the tested concentration. To further address the safety of live bacterial preparations, additional LDH and MTT assays were performed using live *S. thermophilus* DM287 and DM294 (MOI 200) under identical experimental conditions. In the live-cell LDH assay, cytotoxicity values were 0.4% ± 1.2% for DM287 and 0.6% ± 1.0% for DM294, both statistically indistinguishable from the untreated low control under the one-sided directional test (*p* = ns) (Figure 4C). In the live-cell MTT assay, cell viability values were 99.9% ± 10.9% for DM287 and 111.7% ± 2.7% for DM294, neither of which differed significantly from untreated cells under the one-sided directional test (*p* = ns) (Figure 4D). The high control in the LDH assay (∼100% release) again confirmed that the assay performed as expected. Together, the absence of cytotoxicity across both heat-killed and live preparations further supports the *in vitro* biocompatibility of both strains and is consistent with their suitability for further development as candidate oral care ingredients.

### 3.6 Transcriptional downregulation of biofilm-associated genes

To investigate transcriptional responses associated with the observed biofilm inhibition, the expression of *gtfB* and *gtfC* – two glucosyltransferase genes central to sucrose-dependent biofilm formation in *S. mutans* – was measured in co-cultured biofilms at 8 h and 24 h (Figure 5). Gene expression data were analyzed using two-way ANOVA with treatment and biofilm maturation time as factors. A significant main effect of treatment was observed for both *gtfB* and *gtfC* expression across biofilm maturation time points. No significant interaction between treatment and biofilm maturation time was observed for either gene (*p* > 0.05), indicating that the suppressive effect of each strain was consistent across both the early (8 h) and mature (24 h) biofilm stages.

For *gtfB*, at 8 h, co-culture with DM287 reduced expression to 0.663 ± 0.098 relative to the *S. mutans*-only control (34.4% reduction; *p* < 0.05), while DM294 reduced it further to 0.463 ± 0.020 (54.2%; *p* < 0.001). By 24 h, suppression by both strains was sustained. DM287 reduced *gtfB* to 0.503 ± 0.105 (49.7%; *p* < 0.01), and DM294 to 0.463 ± 0.045 (53.7%; *p* < 0.001) (Figure 5A).

A comparable pattern was observed for *gtfC*. At 8 h, expression was reduced to 0.550 ± 0.081 by DM287 (46.1%; *p* < 0.05) and 0.467 ± 0.003 by DM294 (54.2%; *p* < 0.01), and at 24 h to 0.527 ± 0.128 by DM287 (47.8%; *p* < 0.01) and 0.373 ± 0.071 by DM294 (63.1%; *p* < 0.01) (Figure 5B).

DM294 consistently produced stronger suppression than DM287 across both genes and time points, with the most pronounced effect observed for *gtfC* at 24 h. The progressive increase in *gtfB* suppression by DM287 between 8 h and 24 h further suggests that the inhibitory influence on gene expression is maintained throughout biofilm development. As *gtfB* and *gtfC* are key contributors to the production of the extracellular polysaccharide matrix that underpins *S. mutans* biofilm architecture, these results suggest that transcriptional downregulation of glucosyltransferase-associated gene expression may contribute, at least in part, to the observed inhibition of cariogenic biofilm formation.

### 3.7 Comparative inhibition of biofilm formation and EPS production

To further compare the anti-biofilm efficacy of DM287 and DM294 with established reference controls, additional experiments were performed using the commercially utilized probiotic strain *Lacticaseibacillus rhamnosus* GG (LGG) and chlorhexidine (CHX; 0.005%) as comparators. Consistent with previous findings, co-culture with DM287 and DM294 markedly suppressed *S. mutans* biofilm biomass by approximately 96% and 92%, respectively, relative to the *S. mutans*-only control (both *p* < 0.001; Figure 6A). LGG exhibited substantially weaker inhibitory activity (∼52% reduction; *p* < 0.001), while CHX achieved near-complete suppression (∼98%; *p* < 0.001).

To determine whether suppression of *gtfB* and *gtfC* expression was associated with reduced EPS output, EPS content in *S. mutans* biofilms was quantified using the phenol–sulfuric acid method. DM287 and DM294 reduced relative EPS production to approximately 10% and 20% of the control, respectively (both *p* < 0.001; Figure 6B), a magnitude comparable to CHX (∼10%; *p* < 0.001). By contrast, LGG produced only a modest reduction in EPS output (∼12%; *p* < 0.05) despite partially suppressing biofilm biomass. These findings support the interpretation that DM287 and DM294 inhibit EPS-mediated biofilm formation in *S. mutans* and provide functional evidence linking *gtfB*/*gtfC* downregulation to reduced EPS production, through a mechanism that appears distinct from that of LGG, which suppressed biofilm biomass without substantially reducing EPS output.

## 4 Discussion

This study examined the anti-cariogenic potential of two *Streptococcus thermophilus* strains – DM287, isolated from a commercially available yogurt product, and DM294, isolated from the tongue coat of healthy adults – against the principal dental caries pathogens *S. mutans* and *S. sobrinus*. Across seven complementary *in vitro* assays, both strains consistently reduced macroscopic plaque accumulation, suppressed cariogenic biofilm formation without exerting bactericidal effects on the target pathogens, inhibited pathogen adhesion to oral epithelial cells, demonstrated active competitive colonization capacity, showed no cytotoxicity in human oral epithelial cells under either heat-killed or live preparation, and downregulated biofilm-associated virulence gene expression (Squarzanti et al., 2024; Zhang et al., 2024) and reduced EPS production to a degree comparable to chlorhexidine and exceeding that of a conventional probiotic reference strain. These observations should be interpreted as an integrated *in vitro* phenotype rather than as the definitive elucidation of distinct molecular pathways: the activity profile is consistent with multiple, complementary modes of action – biofilm suppression, adhesion interference, competitive colonization, and transcriptional modulation of EPS-synthetic genes – without each having been independently validated as a discrete mechanism. Within this framing, *S. thermophilus* DM287 and DM294 emerge as candidate strains with promising anti-biofilm activity for oral care applications, pending further validation in more physiologically relevant *in vivo* and human-cohort models.

A central finding of this study is the dissociation between biofilm inhibition and planktonic growth suppression observed in Figure 2. While both DM287 and DM294 reduced *S. mutans* and *S. sobrinus* biofilm biomass by more than 95%, co-culture OD600 values were not reduced – and were in fact significantly elevated, reflecting the additive contribution of *S. thermophilus* to total planktonic density. This pattern suggests that the observed effect is not primarily driven by bactericidal or growth-inhibitory mechanisms and is instead more consistent with selective interference with biofilm-specific processes (Sarah Samson et al., 2025). However, it should be noted that OD_600_-based measurements represent total bacterial density and do not allow discrimination between individual species in co-culture systems. Therefore, while the present data are consistent with a growth-independent anti-biofilm effect, they do not definitively exclude the possibility of species-specific growth inhibition. The pathogenicity of mutans streptococci is fundamentally biofilm-dependent: it is the structured, EPS-scaffolded plaque – rather than planktonic cell density – that drives the acidogenic microenvironment responsible for enamel demineralization (Chen et al., 2022; Zhu et al., 2023). A probiotic that suppresses biofilm architecture without killing the pathogen may therefore represent a targeted strategy for modulating cariogenic biofilms while minimizing disruption to the overall microbial community (Rebelo et al., 2023). This non-bactericidal mode of action is particularly relevant for dentifrice formulation, where daily use demands a safety and selectivity profile incompatible with indiscriminate antimicrobial activity (Hwang et al., 2025). Such competitive interference may contribute to the observed reductions in *gtf* expression and subsequent biofilm development. The gene expression data in Figure 5 provide molecular support for this anti-biofilm phenotype. Co-culture with DM287 or DM294 significantly downregulated *gtfB* and *gtfC* expression in *S. mutans* biofilms at both the early (8 h) and mature (24 h) stages, with reductions ranging from approximately 34%–54% at 8 h and 50%–63% at 24 h depending on the strain and gene. As *gtfB* and *gtfC* encode the glucosyltransferases responsible for synthesizing the sucrose-dependent extracellular polysaccharides that form the primary structural scaffold of *S. mutans* biofilms, their downregulation offers a plausible molecular mechanism linking probiotic co-culture to the profound biofilm suppression observed in Figure 2. DM294 consistently produced stronger transcriptional suppression than DM287, and the progressive increase in *gtfB* inhibition between 8 h and 24 h suggests that the effect is maintained – and possibly amplified – as biofilms mature. These findings suggest that downregulation of glucosyltransferase-associated gene expression may contribute, at least in part, to the observed anti-biofilm activity (Ahn et al., 2005). EPS quantification by phenol–sulfuric acid assay further confirmed that both DM287 and DM294 significantly reduced EPS production in *S. mutans* biofilms, supporting the interpretation that these strains interfere with glucosyltransferase-mediated EPS synthesis at the functional level.

Importantly, the additional comparative experiments performed using *Lacticaseibacillus rhamnosus* GG (LGG) and chlorhexidine further contextualized the magnitude of the observed anti-biofilm activity. Both DM287 and DM294 suppressed *S. mutans* biofilm formation and EPS production to a significantly greater extent than LGG and achieved effects comparable to those observed with chlorhexidine (Figures 6A,B). These findings suggest that the anti-biofilm activity of the two *S. thermophilus* strains is not only statistically significant but also biologically meaningful when benchmarked against established probiotic and antimicrobial reference controls.

The macroscopic plaque data from Figure 1 extended the biofilm findings to a more physiologically representative system. Using orthodontic stainless steel wires, both strains achieved approximately 75%–80% reduction in plaque mass against both pathogens (Fijan, 2023). This level of inhibition in a three-dimensional plaque model carries greater translational relevance than standard 96-well biofilm assays alone, as it reflects the ability of the probiotic to suppress plaque under conditions closer to those encountered in the oral cavity during dentifrice use (Sarah Samson et al., 2025). The comparable efficacy of DM287 and DM294 across both pathogen species in this assay suggests that plaque suppression is a robust, strain-conserved property.

The epithelial adhesion inhibition data (Figure 3) further demonstrated that both strains can interfere with pathogen colonization at the mucosal interface, with displacement consistently more effective than protection in most conditions (Squarzanti et al., 2024). Against *S. sobrinus* in particular, displacement-mode inhibition (∼75%) far exceeded protection-mode inhibition (∼33%), suggesting that both strains can actively dislodge pre-adhered pathogens rather than merely occupying adhesion sites in advance (Zymovets et al., 2025). This is mechanistically coherent with the competitive colonization data in Figures 3E,F, which showed that both strains significantly increased their surface attachment in pathogen-conditioned environments, with fold changes ranging from 1.13 to 2.01. The strain-specific differences in competitive affinity – DM287 competing more effectively against *S. mutans* and DM294 against *S. sobrinus* – may reflect differences in surface adhesin repertoire or co-aggregation properties between the two strains, though the molecular basis warrants further investigation. These findings further support the possibility that competitive surface colonization by *S. thermophilus* may limit the early establishment of cariogenic pathogens and indirectly reduce EPS-driven biofilm maturation.

From a product development perspective, the selection of *S. thermophilus* as an oral care probiotic candidate is supported by a convergence of practical and regulatory considerations. Oral probiotic research has to date been dominated by *Lactobacillus* species and *Streptococcus salivarius* strains K12 and M18, which have well-characterized clinical efficacy profiles but were originally isolated from the human oral cavity rather than established food production pipelines (Mato et al., 2024). *S. thermophilus*, by contrast, has been consumed at scale for decades as a yogurt starter culture, accumulating a safety record that underpins both its GRAS and QPS regulatory designations (Kapse et al., 2024). This existing regulatory infrastructure substantially reduces the burden of safety substantiation required for novel ingredient applications (Panel, 2024). Furthermore, *S. thermophilus* is among the very few probiotic species for which both food-grade production scalability and oral mucosal safety are generally regarded as established – a combination that is rare among candidate oral probiotics and directly relevant to oral care formulation development, where ingredient safety, manufacturing feasibility, and regulatory compliance must be simultaneously satisfied (Allouche et al., 2024).

The cytotoxicity data from Figure 4 indicate no evidence of cell membrane damage or metabolic suppression in KB oral epithelial cells at the tested concentration. Notably, the consistent absence of cytotoxicity across both heat-killed and live-cell conditions confirms that the biocompatibility of these strains is a stable property, independent of metabolic activation. The safety profile is further substantiated by the ecological origins of the isolates: DM287 was obtained from a commercial yogurt product with an established history of safe consumption, while DM294 was isolated from the healthy oral mucosa of an adult, indicating inherent compatibility with the human oral environment. These strain-level findings align with the global safety record of *S. thermophilus*, which holds GRAS and QPS status based on extensive human consumption in fermented dairy products (Kapse et al., 2024; Panel, 2022). While these results establish a robust *in vitro* safety foundation, further *in vivo* and clinical studies will be necessary to confirm safety and efficacy in humans.

The present study has several limitations that should be interpreted in the context of existing literature. Although species identification was confirmed by 16S rRNA gene sequencing and both strains were deposited in the Korean Collection for Type Cultures (KCTC), additional strain-level characterization using methods such as RAPD, MLST, or whole-genome sequencing would further strengthen discrimination between isolates and provide deeper insight into strain-specific functional traits. The experimental framework was entirely *in vitro*, and whether the observed effects translate to the compositional complexity of the human oral cavity remains an open question – a challenge common to early-stage probiotic characterization studies (Sarah Samson et al., 2025). The wire model and biofilm assays relied on sucrose-supplemented monoculture or dual-culture systems, which do not fully recapitulate the compositional and structural complexity of natural dental plaque (Sangha et al., 2024). The number of biological replicates (*n* = 3) is consistent with exploratory *in vitro* characterization, as applied across comparable studies in this field, but limits statistical power to detect smaller effect sizes. Regarding mechanism, the gene expression data partially addressed the molecular basis of biofilm inhibition – specifically the downregulation of *gtfB* and *gtfC* – yet whether organic acid production, competitive co-aggregation, or additional factors contribute remains to be determined through targeted follow-up investigation.

Confocal laser scanning microscopy (CLSM) imaging with EPS staining would additionally provide structural and spatial context that the current data cannot offer. Future studies incorporating multispecies oral biofilm models, CLSM imaging with EPS-specific staining, and direct glucosyltransferase activity measurements would provide a more comprehensive understanding of the mechanisms underlying biofilm suppression. The stability and viability of DM287 and DM294 under dentifrice formulation conditions remain to be assessed; compatibility testing – identified as an essential prerequisite for probiotic dentifrice development – will be necessary before formulation work can proceed (Tonguc-Altin et al., 2024). Notwithstanding these limitations, the multi-assay evidence base presented here compares favorably with prior *in vitro* characterization of oral probiotic candidates. Previous studies reporting competitive exclusion or biofilm suppression by *Lactobacillus* strains have generally relied on single-assay designs; the seven-assay panel employed here – spanning plaque mass, biofilm biomass, planktonic viability, adhesion inhibition, competitive colonization, cytotoxicity, virulence gene expression, and EPS quantification – provides a more systematic functional profile. The non-bactericidal mode of action observed for DM287 and DM294 is consistent with the microbiome-modulating paradigm increasingly prioritized over broad-spectrum antimicrobial approaches in caries prevention (Beattie, 2024). In addition, direct bactericidal activity was not assessed using viability-based species-specific methods, which limits definitive interpretation of the underlying mechanism. Although the present study incorporated species-specific qPCR analysis to assess pathogen abundance under co-culture conditions, additional approaches such as selective plating or live/dead viability assays may further clarify the precise contribution of bactericidal versus biofilm-specific mechanisms. Finally, all findings were obtained under *in vitro* conditions, and *in vivo* and clinical validation will be required before translation to oral care applications can be considered.

*Streptococcus thermophilus* DM287 and DM294 emerge as candidate oral probiotic strains with promising anti-biofilm potential for caries prevention applications.

## Data Availability

The data supporting the findings of this study are available within the article. The bacterial strains characterized in this study have been deposited at the Korean Collection for Type Cultures (KCTC) under accession numbers KCTC16622BP (*S. thermophilus* DM287) and KCTC16550BP (*S. thermophilus* DM294). Further inquiries can be directed to the corresponding author.
