# Supplementary material for: Streptococcus thermophilus DM287 and DM294 as candidate oral probiotic strains with anti-biofilm activity against cariogenic pathogens
Source: Front Microbiol. 2026 Jun 23;17:1852167. doi: 10.3389/fmicb.2026.1852167 (PMC13337710; doi:10.3389/fmicb.2026.1852167)
Supplement: Supplementary file 1 [file Image_1.pdf]

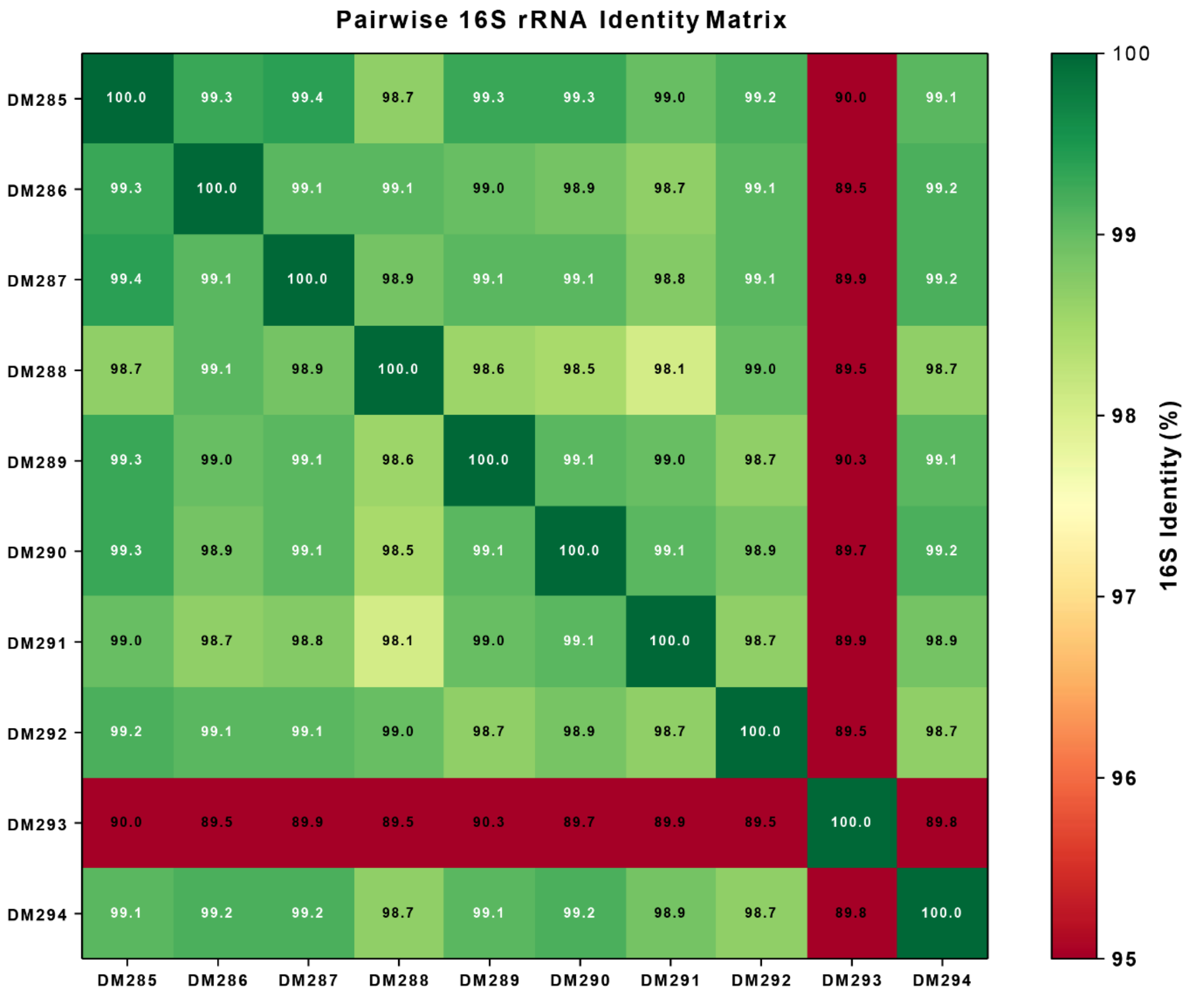

Supplementary Figure S1. Pairwise 16S rRNA sequence identity matrix of the initial *S. thermophilus* screening panel.

Pairwise 16S rRNA gene sequence identities (%) among ten candidate isolates (DM285–DM294) recovered from tongue coat specimens obtained through the Appletree Oral Biobank and from a commercially available yogurt product. Isolates were identified by PCR amplification using universal primers 27F/1492R, followed by NCBI BLAST homology analysis. Color scale indicates percent identity from 95% (red) to 100% (dark green). All isolates except DM293 shared ≥98.7% pairwise identity and were confirmed as *Streptococcus thermophilus* (≥99% identity to reference sequences). DM293 exhibited markedly lower identity to all other isolates (~89–90%), consistent with classification as a distinct species, and was excluded from downstream analysis. Strains DM287 and DM294 were selected for functional characterization based on confirmed species identity, distinct ecological origins (yogurt-derived vs. human tongue coat-derived, respectively), and representation of inter-strain sequence diversity within the panel.
